# Segment Regeneration of an Earthworm I: Formation of the Body Wall Tissues from Injury to Recovery

**DOI:** 10.3390/life16010119

**Published:** 2026-01-13

**Authors:** Gabriella Laura Tóth, Edit Pollák, Anita Erdélyi, Eszter Várhalmi, Zsolt Pirger, István Fodor, László Molnár

**Affiliations:** 1 Ecophysiological and Environmental Toxicological Research Group, HUN-REN Balaton Limnological Research Institute, 8237 Tihany, Hungary; toth.gabriella@blki.hu (G.L.T.); pirger.zsolt@blki.hu (Z.P.); fodor.istvan@blki.hu (I.F.); 2 Department of Neurobiology, Institute of Biology, Faculty of Natural Sciences, University of Pécs, 7624 Pécs, Hungary; peditmail@gmail.com (E.P.); erdelyianita1993@gmail.com (A.E.); esztervarhalmi@gmail.com (E.V.)

**Keywords:** wound healing, dedifferentiation, histogenesis, regeneration, collagen

## Abstract

Segment regeneration in earthworms is a remarkable example of postembryonic morphogenesis, yet its fidelity and cellular mechanisms remain incompletely understood. The present study investigated posterior segment regeneration in adult specimens of the earthworm model *Eisenia andrei* from wound closure to the 5th postoperative week using anatomical, histological, and ultrastructural approaches. Rapid wound closure occurred through fusion of the cut edges of the body wall and midgut without direct involvement of coelomocytes. The regeneration blastema consisted of dedifferentiated epithelial and muscle cells, innervated by fibers from the last intact ventral nerve cord ganglion. Coelomocytes accumulated in the last intact segments and were primarily involved in debris clearance. Notably, early regenerating tissues lacked collagen fibers, which appeared only after the third postoperative week and remained sparse until the fifth week, whereas original segments exhibited intense, region-specific collagen deposition. Transmission electron microscopy revealed characteristic cytological changes in distinct stages of body wall regeneration, including muscle dedifferentiation and the emergence of collagen-producing fibroblasts. These findings indicate that early cell migration, proliferation, and orientation in the blastema proceed independently of collagen and that collagen functions as a delayed structural scaffold, supporting tissue integrity without impeding regeneration. Importantly, no scar formation was observed between old and new tissues, resembling scarless fetal wound healing. Overall, we clarified previously controversial cellular mechanisms and propose a new, comprehensive model for the early stages of segment regeneration. Our results highlight that coordinated dedifferentiation, spatiotemporal extracellular remodeling, and delayed collagen deposition underlie effective, scar-free regeneration in earthworms, offering insights into conserved mechanisms of regenerative repair across metazoans and potential strategies for enhancing tissue regeneration in mammals.

## 1. Introduction

Reparative regeneration (shortly regeneration) is an enigmatic process of some metazoan species that can restore their lost body parts. During the last centuries, animals with regeneration ability were identified by the scientific community. The main finding of the detailed investigations was that the regeneration ability of animals does not exhibit phylogenetic dependence, as outstanding regenerative capacity is characteristic for cnidarians, platyhelminthes, annelids, and some vertebrates, such as newts and salamanders (reviewed by [1,2]). Moreover, sometimes in the same animal taxa, there are species with high regeneration capacity and no regeneration ability as well. For example, cnidarian polyps survive the loss of over 80% of their bodies and then regenerate the missing structures, while medusae do not tolerate tissue loss. They do not regenerate; instead, they die [3,4]. A similar situation is characteristic of the annelid earthworm and leeches [4], or the amphibian salamanders and frogs [5]. Based on the comprehensive analysis of the regeneration ability of invertebrate and vertebrate species, the importance of the lifestyle of a species and the influences of ecological parameters were suggested [6].

It has also been revealed that regeneration is a special form of postembryonic morphogenesis, which is genetically governed [7,8], but it is influenced by several internal and external factors [9]. There is growing evidence for the neural dependence of regeneration. The presence of intact or hurt neural processes in the damaged structure is necessary for its regular reorganization. It was recognized that both electric signals and chemical compounds of the neurons had a great impact on the formation of the regeneration blastema from which new tissues were differentiated [10,11,12,13,14]. Stimulatory effects of some neurotransmitters, like gamma aminobutyric acid [15,16] and serotonin [17,18,19], on neuronal development, nerve growth, and axon regeneration were demonstrated in distinct species. During the last two decades, the influence of the endocrine system on regeneration was also investigated, and the experimental results proved hormonal dependence in some phases of the reorganization of the tissues (reviewed by ref. [20]). Nowadays, a hot topic in regeneration biology is the influence of the immune system on wound closure, regeneration blastema formation, and morphogenesis of the new tissues in vertebrates [21,22,23,24,25]. However, it was not labeled as an immune system-influenced process. Ref. [26] showed that the extraction of the coelomocytes (immune cells of the earthworms) strongly inhibited segment regeneration. Later, coelomocytes were found to contain high amount of riboflavin, which is thought to act as a stimulating factor in various cellular processes, such as proliferation and differentiation [27]. Given this, the presence of coelomocytes with riboflavin was concluded to be essential for regular regeneration of the nervous system [27].

Environmental factors are also known to significantly influence segment regeneration. Studies have shown that exposure to cadmium enhances regenerative ability at room temperature, but low temperatures inhibit both regeneration and reproduction in *E. andrei*, even though immune cell renewal remains largely unaffected [28]. Similarly, research on *E. fetida* demonstrated that worms maintained at temperatures below 24 °C or above 30 °C exhibit slower regeneration rates, highlighting the critical role of temperature in regulating regenerative processes [29]. Retinoic acid has been shown to delay and disrupt regeneration by reducing the expression of the *Sox2* gene and protein, which are both crucial for blastema formation [30]. The effects of environmental pollutants on regeneration have also been explored. Exposure to carbamate pesticides, such as metolcarb and fenoxycarb, caused significant toxicity and impaired tail regeneration in *E. fetida* [31]. In contrast to environmental toxins, terahertz (THz) pulse irradiation has been shown to enhance regeneration. Single-cycle THz pulses significantly promoted cell proliferation, histogenesis, and organogenesis in excised *E. andrei*, resulting in the regeneration of more segments and more robust development of the central nervous system and blood vessels [32].

Although our knowledge of regeneration intensely developed during the last decades, some questions remain unanswered by the scientific community. One of the most prominent questions is the fidelity of regeneration: are the regenerated structures identical to the lost ones in terms of structure and function, and if so, to what extent? To answer this, convenient models are necessary that have a simple body plan, and the histological characteristics of both damaged and renewing tissues can easily be investigated by microscopical methods. Epimorphic regeneration of planarians and morphallactic regeneration of hydras, well-established models of regeneration biology, are considered highly faithful regenerations, capable of recreating both structure and function accurately [33,34,35]. Some earthworm species are also eligible to be model animals of regeneration, since the kinetics of their segment restitution have already been investigated in detail [36,37,38]. However, it had been recognized that during the segment regeneration, no scar was formed between the original and renewing tissues, and the background of its histological phenomenon was not identified yet [39]. Moreover, there is a long-standing debate about the fidelity and the early-stage mechanisms of segment regeneration in earthworms.

The aim of the present study was to investigate the anatomical, histological, and ultrastructural changes during both the early and late phases of posterior segment regeneration of the earthworm model *E. andrei*. To accomplish our aim, we removed the last 25 tail segments of mature specimens and first monitored the anatomical differences (e.g., metanephridia, dorsal pores, ganglia) at the 3rd and 6th postoperative hours. Next, we examined the course of wound closure and the composition of the regeneration blastema. Moreover, we also investigated the histological and histochemical alterations (e.g., collagen deposition) at the 1st, 7th, 21st, and 35th postoperative days. Finally, we examined the precise ultrastructural changes, including collagen fibers and muscle dedifferentiation, at the 1st, 3rd, 6th, and 24th postoperative hours and the 1st, 3rd, and 5th postoperative weeks.

## 2. Materials and Methods

### 2.1. Experimental Animals

Breading stocks of *Eisenia andrei* (Clitellata, Lumbricidae) were maintained on a substrate composed of a 6:3:1 weight ratio mixture of potting soil (pH = 6.5), sphagnum peat, and quartz sand, supplemented with 1 g of calcium carbonate per kilogram of soil. The substrate was moistened with cold tap water, and the animals were fed ad libitum with alfalfa pellets soaked in tap water. Worms were housed at 23 °C (±1 °C) under a 12 h:12 light/dark regime. Sexually matured (clitellated) specimens were selected for the experiments and kept on a tap water-moistened paper towel for two days to remove gut contents.

### 2.2. Surgical Intervention

After anesthetization with ice-cold carbonated water, the last 25 tail segments of 15 animals for each time point of microscopic investigations were surgically removed at the level of the body furrow using sharp surgical scissors. The wound surface was examined under a dissecting microscope, and only specimens with a straight-line wound boundary were selected for further investigation. After the surgical intervention, worms were kept on a tap water-moistened paper towel, and their physiological state and the process of wound closure were monitored every hour until the 6th postoperative hour, by which time the wounds of most animals had fully closed. Animals showing signs of physiological weakness (slow movement; collapsed, swollen, or sometimes bloodshot segments) or abnormalities in wound closure were excluded from the experiments (7–12% of the total number).

### 2.3. Histological and Histochemical Investigations

Samples of the transected segments, together with 6–8 adjacent segments, were collected from freshly operated, deeply anaesthetised worms at the 1st, 3rd, and 6th postoperative hours and subsequently at the 1st, 3rd, 7th, 21st, and 35th postoperative days. At each point in time, 10 animals were sacrificed for sample collection. In an experimental frame, 80 animals were used for microscopic investigations, and the experiments were repeated three times.

For conventional histology and collagen histochemistry, the isolated body parts were fixed in a freshly prepared formalin–acetic acid mixture (6 mL 38% formaldehyde, 1 mL glacial acetic acid, and 18 mL distilled water) at room temperature for 5–7 days. After fixation, tissue processing (washing, dehydration, and paraffin embedding) was performed according to standard histological protocols of the Histological and Histochemical Methods handbook (see ref. [40]). Before embedding, the paraffin-infiltrated tissue blocks were oriented under a stereo binocular microscope to determine the optimal sectioning planes. Serial sections of 10 µm thickness were prepared using a Reichert rotary microtome and mounted onto slides.

Standard haematoxylin and eosin (H&E) staining was used for histological examinations. To investigate the organization of the extracellular matrix, including new collagen deposition during wound healing and tissue remodeling, Picrosirius Red (PSR) staining, as suggested by [41], was applied. Briefly, slides were incubated for 20 min at 25 °C in PSR staining solution (100 mg of Direct Red 80 (# 365548, Merck, Budapest, Hungary) in 100 mL of saturated picric acid), followed by rinsing with 0.5% acetic acid in distilled water. This method proved to be more sensitive in identifying collagen bundles and fibers than the traditional polarization microscopy for collagen histochemistry. Both H&E- and PSR-stained sections were dehydrated through a graded ethanol series, cleared in xylene, and cover-slipped with DPX mounting medium (Apollo Scientific, Denton, UK).

All sections were examined using a Zeiss Axioplan microscope, employing bright-field illumination for H&E-stained sections and fluorescence with an FITC filter cube (Excitation: 450–490 nm, Emission: 500–550 nm) for PSR-stained sections, following the method described by ref. [42].

### 2.4. Electron Microscopy

The same body region of regenerating animals was isolated at distinct time points (1st, 3rd, 6th, and 24th postoperative hours and 1st, 3rd, and 5th postoperative weeks) and fixed in a modified Karnovsky solution (4% paraformaldehyde and 2.5% glutaraldehyde in 0.1 M cacodylate buffer, pH 7.2) for 2 h at 0 °C. At each point in time, original and regenerated tissue samples were isolated from three animals. During thorough washing (four changes of ice-cold 0.2 M cacodylate buffer, pH 7.2, for 2 h), small tissue samples were dissected from the segments. Since the most advanced part of the regenerating blastema (when present) was connected to the ventromedial region of the segments, where the ventral nerve cord is located, this region was isolated for further processing. The samples were post-fixed in ice-cold 1% osmium tetroxide in cacodylate buffer for 2 h, washed in 50% ethanol for 5 min, and dehydrated through an ascending ethanol series from 70% to 100%. After immersion in propylene oxide, the samples were infiltrated with Durcupan ACM Araldite resin (Merck, Budapest, Hungary). Following resin polymerization, semithin sections (0.1 µm) were cut using a Reichert ultramicrotome and stained with 0.1% toluidine blue to identify regions of interest for further analysis. Ultrathin sections were then cut with a diamond knife, mounted on nickel grids, contrasted with saturated uranyl acetate in distilled water and Reynolds’ lead citrate, and examined using a JEOL 1200EX II transmission electron microscope (JEOL Europe SAS, Croissy Sur Seine, France).

## 3. Results

### 3.1. Anatomical Alterations After the Segment Ablation

**Figure 1:**
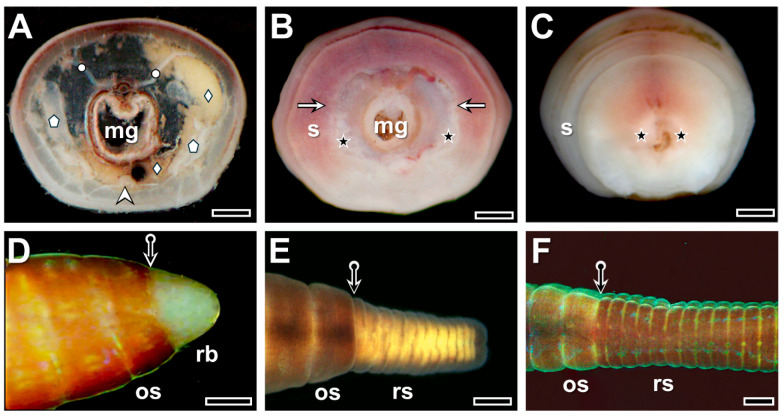
Anatomical characteristics of the transected segment of *E. andrei* from the surgical intervention (**A**), through the 3rd (**B**) and 6th (**C**) postoperative hours, and the formation of the regeneration blastema at the 1st postoperative week (**D**), as well as new segments at the 3rd (**E**) and 5th (**F**) postoperative weeks. Labeling: mg: midgut, arrowhead: ventral nerve cord, circle: dissepimental muscle, deltoid: coelomocytes, pentagon: metanephridium, asterisk: white tissue at the wound boundary, arrow: boundary of the transected body wall, bullet arrow: boundary of the original segments (os) and regeneration blastema (rb) or regenerating segments (rs). Scale bars: (**A**–**C**): 250 μm, (**D**–**F**): 500 μm.

Correct ablation of tail segments resulted in smooth surfaces of the transected organs and preserved the integrity of the dissepiment (Figure 1A), allowing free-floating coelomocytes washed out from the injured segment to accumulate only in the anterior intact segments. The surgical intervention imposed strong physiological stress on the animals. They lost body fluids and coelomocytes during both anaesthesia and the operation, but most began to recover from the shock by the 1st postoperative hour, with only about 8–10% exhibiting physical weakness (and subsequently excluded from the experiments). Contraction of both circular and longitudinal muscles brought the body wall closer to the transected midgut tissues by the 3rd postoperative hour, when the formation of a loose, white tissue at the wound boundaries became a characteristic morphological change (Figure 1B). By the 6th postoperative hour, the wound was completely closed with a thin layer of white tissue plug situated between the body wall and midgut tissues (Figure 1C).

By the 1st postoperative week, a white tissue mass—the regeneration blastema (RB)—formed and attached to the transected segment (Figure 1D). Within the transparent RB, no blood vessels, coelomocytes, or any anatomical markers of prospective segmentation were yet observed. By the 3rd postoperative week, several defined segments with a transparent prospective body wall developed, containing a high number of yellow coelomocytes (Figure 1E). By the 5th postoperative week, the number of regenerated segments was sometimes equal to, and sometimes nearly equal to, that of the ablated segments; however, their size and pigmentation remained significantly different from those of the original segments (Figure 1F). In the ventral part of the new segments (not shown), the coelomic sacs were densely filled with coelomocytes.

### 3.2. Histological and Cytological Characteristics of the Intact Body Wall Tissues

**Figure 2:**
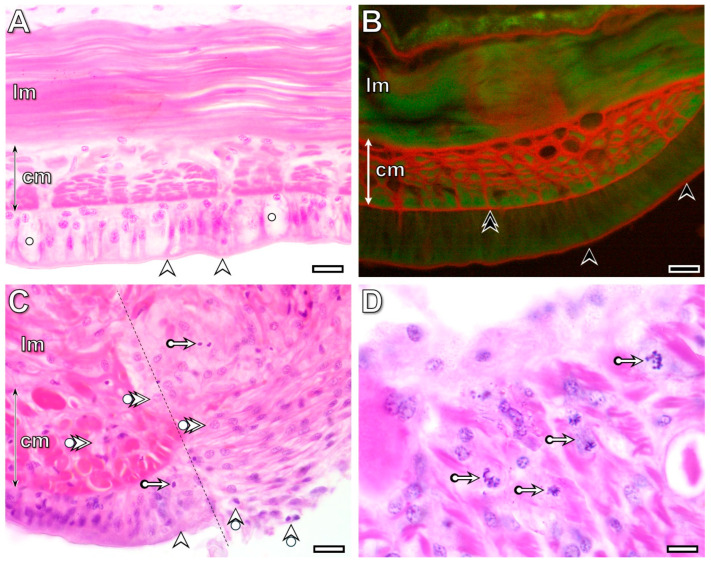
Characteristic histological properties of the intact (**A**,**B**) and transected ventral body wall at the 3rd (**C**) and 6th (**D**) postoperative hours. Collagen fibers are labeled with picrosirius red staining (**B**). Note that dedifferentiated muscle cells occur in both the circular and longitudinal muscle layers, and some of them exhibit mitotic activity. Labeling: cm: circular muscles, lm: longitudinal muscles, dotted line: site of the transection, arrowhead: cuticle, double arrowhead: basement membrane, bullet arrowhead: dedifferentiated epithelial cell, bullet double arrowhead: dedifferentiated muscle cell, bullet arrow: mitotic cell. Scale bars: (**A**,**B**): 100 μm, (**C**): 25 μm, (**D**): 10 μm.

**Figure 3:**
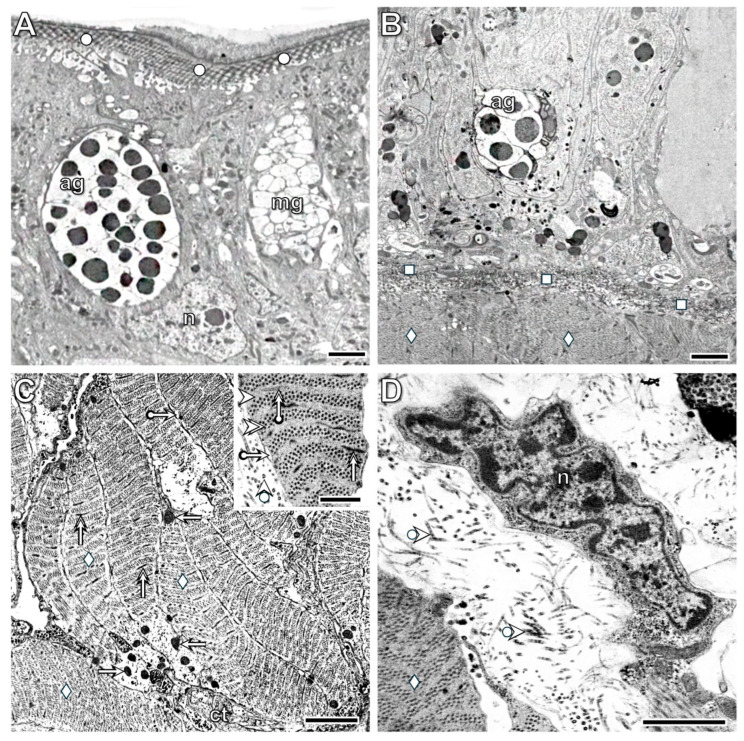
Distinct epithelial cell types are tightly attached to their neighbors, and no extended extracellular spaces are observed (**A**). The muscle fibers of both the circular (**B**) and longitudinal (**C**) muscle layers are surrounded by thin connective tissue containing some collagen fibers. Among muscle fibers, a few fibroblasts, surrounded by collagen fibrils, are present (**D**). Labeling: ag: albumen gland, ct: connective tissue, mg: mucous gland, n: nucleus, circle: cuticle, square: basement membrane, deltoid: muscle fiber, arrowhead: I-band, double arrowhead: A-band, arrow: mitochondrion, bullet arrowhead: collagen fibril, bullet arrow: peripheral zone of the sarcoplasmic reticulum, double bullet arrow: tubular element of the sarcoplasmic reticulum, double arrow: Z-rod. Scale bars: (**A**–**C**): 2 μm, insert of (**C**): 500 nm, (**D**): 1 μm.

The fourth and fifth intact segments from the transected one of the freshly operated animals were used as controls for light and electron microscopic investigations. In these segments, the pseudostratified columnar epithelium consisted of tightly connected, distinct cell types, including small basal cells, columnar supporting cells, and gland cells, covered by a collagen-rich cuticle (Figure 2A and Figure 3A). All cells were attached to the collagen-rich basement membrane, forming a barrier between the epithelium and circular muscles (Figure 2B and Figure 3B). Thin connective tissue septa containing some collagen fibers were present between the circular and longitudinal muscle fibers. In longitudinal sections, they were easily identified around the circular muscle fibers and at the boundary between the muscle layers (Figure 2B). Contractile elements occupied most of the muscle fiber volume and were arranged to form alternating A- and I-bands. Within the I-bands, Z-rods and tubular elements of the sarcoplasmic reticulum exhibited a narrow vesicle-like peripheral zone (Figure 3C). Fibrocytes, surrounded by a wider extracellular space containing some collagen fibers, were rarely observed among the muscle fibers (Figure 3D).

### 3.3. Wound Healing and RB Formation Are Supported by a Dynamic Dedifferentiation of Body Wall Tissues

Due to the transection of the segment, several morphological modifications of distinct tissues—such as swelling of the muscle layers of both the body wall and the midgut, disorganization of the injured body wall and midgut epithelium, and the appearance of roundish cells near the cut surfaces—were observed in samples fixed at the 3rd postoperative hour (Figure 2C). By the 3rd postoperative hour, injury-induced tissue stress mechanisms were activated in all organs. These processes stimulated the rapid dedifferentiation of cells in both the circular and longitudinal muscle layers and in the epithelium at the wound boundary, extending into deeper neighboring tissue regions by the 6th postoperative hour. A high number of mitotic cells appeared among the dedifferentiated muscle cells in some specimens (22% of the experimental animals), though not in all (Figure 2D). The morphology of the dedifferentiated epithelial and longitudinal muscle cells differed markedly. Dedifferentiated epithelial cells became slightly elongated with a large nucleus and pale-stained cytoplasm, whereas dedifferentiated muscle cells were roundish with pale cytoplasm and a large nucleus. In contrast, distinguishing dedifferentiated circular muscle cells from epithelial cells was problematic using light microscopy because of their similarly elongated morphology (Figure 2C).

**Figure 4:**
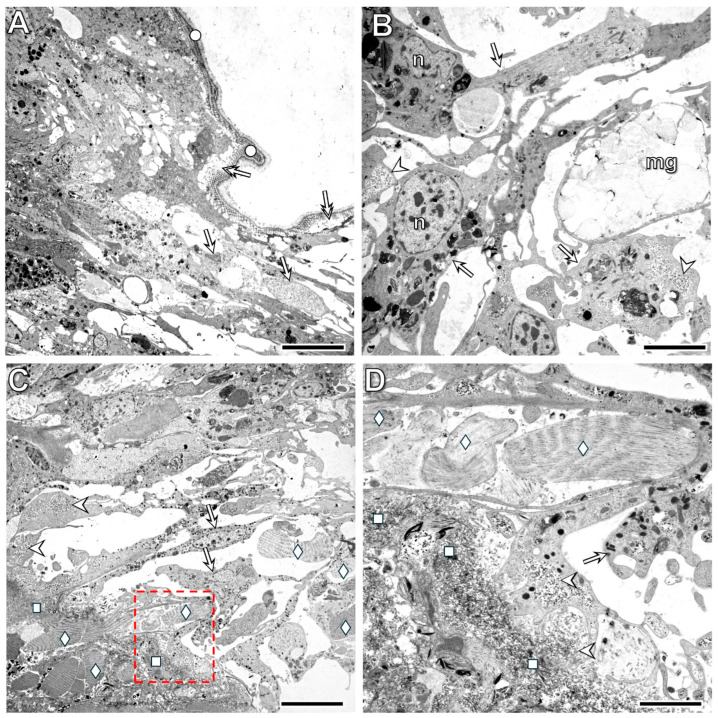
Rapid disorganization (dedifferentiation) of the epithelial cells located near the transection site (right side of (**A**)) is a characteristic change. Detached gland cells displayed characteristic signs of anoikis (**B**). Several epithelial cells maintain their connections to the basement membrane, although disruption of the membrane is also evident. Ingression of muscle fibers into the epithelial layer (**C**) and its enlargement in (**D**) is another characteristic alteration. Labeling: circle: cuticle, arrow: dedifferentiated cell, double arrow: disruption of the cuticle and epithelial connection, mg: mucous gland cell, n: nucleus, deltoid: muscle fiber, square: basement membrane, arrowhead: intracellular deposition of collagen fibrils. Scale bars: (**A**,**C**): 10 μm, (**B**): 5 μm, (**D**): 2 μm.

Ultrastructural investigations supported the histological observations; rapid, pronounced disorganization was seen in the body wall epithelium near the transection. In addition to epithelial cells, some muscle fiber-like structures appeared beneath the cuticle (Figure 4A). Morphological alterations in certain epithelial cells exhibited cytological signs of dedifferentiation. They lost their connections to neighboring cells, supporting cells transformed into pseudopod-bearing cells, and accumulations of osmiophilic granules (lysosome-like structures) and collagen filament-containing vesicles were present. In contrast, detached gland cells displayed characteristic signs of anoikis, such as a roundish shape, swelling, and degeneration of cytoplasmic components (Figure 4B). Detachment of the cuticle from dedifferentiated epithelial cells was also characteristic. Although some epithelial cells remained attached to the basement membrane, their disruption had occurred as well, leading to the ingression of muscle fibers among the epithelial cells (Figure 4C,D).

**Figure 5:**
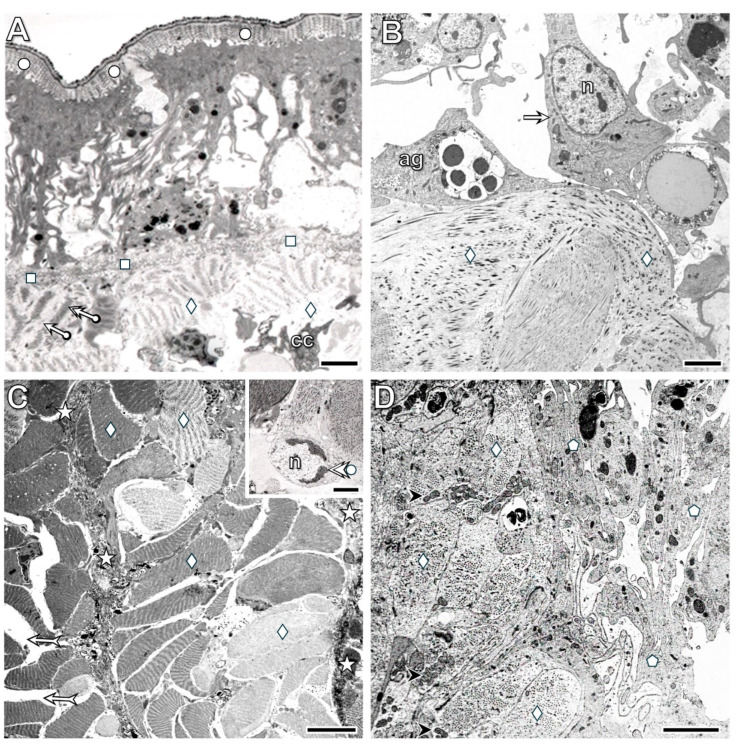
The image in (**A**) was taken from the region farther from the transection, whereas (**B**) shows the region closer to the transection by the 3rd postoperative hour. In the farther region, both the epithelial cells and circular muscle fibers are dedifferentiated, but the basement membrane appears to remain intact. Near the transection, no basement membrane is present between the dedifferentiated epithelial cells and muscle fibers. Two distinct forms of longitudinal muscle ultrastructure are shown in (**C**,**D**) (see detailed information in the text). Among the clear muscle fibers, apoptotic forms were also observed (inset of (**C**)). Labeling: cc: coelomocyte, ag: albumen gland, n: nucleus, circle: cuticle, square: basement membrane, arrow: dedifferentiated supporting cell, bullet arrow: I-band, bullet double arrow: A-band, deltoid: muscle fibers, forked arrow: extended extracellular space, pentagon: dedifferentiated muscle fibers, double bullet arrowhead: apoptotic nucleus, asterisk: collagen deposition. Scale bars: (**A**,**C**): 5 µm, (**B**,**D**): 2 μm, insert: 1 μm.

**Figure 6:**
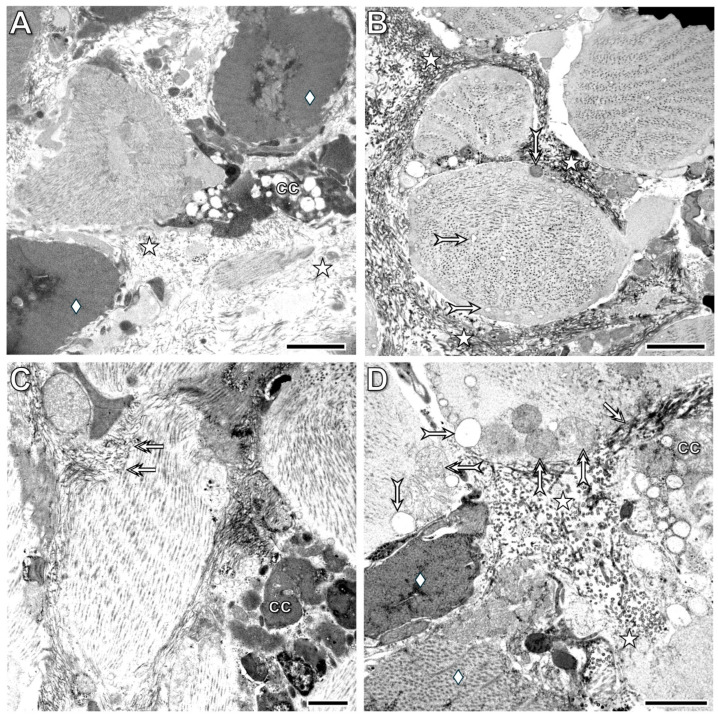
Distinct types of dedifferentiating muscle fibers found in the injured segment distant from the transection. Note that during muscle fiber dedifferentiation, in addition to the disorganization of the A- and I-bands (**A**), the realignment of the sarcoplasmic reticulum (**B**) and the translocation of swollen mitochondria (**B**,**D**) are also characteristic steps. Close connections between muscle fibers and coelomic cells were frequently observed (**A**,**C**,**D**). A portion of the deposited collagen fibers is produced by the dedifferentiated muscle fibers (**C**,**D**). Labeling: cc: coelomic cell, deltoid: muscle fiber, asterisk: deposition of the collagen fibrils, forked arrow: vesicle of the sarcoplasmic reticulum, double arrow: exocytosis of the collagen fibrils from the muscle fiber, forked double arrow: swollen mitochondrion. Scale bars: (**A**,**B**): 2 μm, (**C**,**D**): 1 μm.

By the 3rd postoperative hour, a more characteristic realignment of the body wall tissues was visible in the transected segment. Farther from the cut site, both epithelial cells and circular muscle fibers showed clear cytological signs of dedifferentiation. Epithelial cells lost both their intercellular junctions and their attachments to the basement membrane and transformed into amoeboid-like cells with cellular processes. The density of muscle fibers decreased due to rapid disorganization of their contractile machinery, including both A- and I-bands. At this point, the integrity of both the cuticle and the basement membrane appeared to be maintained (Figure 5A). In contrast, near the transection, the basement membrane was absent from the dedifferentiated epithelial cells and muscle fibers (Figure 5B). Transformation of the muscle fibers in the longitudinal layer was also observed, but two distinct patterns occurred in different specimens. In some, dedifferentiating (clear) muscle fibers were located among undifferentiated (dark) fibers, and small deposits of collagen fibers appeared both between the muscles and at the wound boundary (Figure 5C). Some muscle fibers lost their junctions with neighboring fibers, resulting in a wide, collagen-filled extracellular space. Among the clear muscle fibers, apoptotic forms (insert of Figure 5C) were also detected. In dedifferentiating muscle fibers, disorganization of the A- and I-bands (Figure 6A), realignment of the sarcoplasmic reticulum (Figure 6B), and translocation of swollen mitochondria (Figure 6B,D) were characteristic. Part of the deposited collagen fibers originated from the dedifferentiated muscle fibers (Figure 6C,D). Close interactions between muscle fibers and coelomic cells were frequently observed as well (Figure 6A,C,D). In other specimens, tightly attached dedifferentiated muscle fibers were confined to the muscle layer, and nearby dedifferentiated cells with irregular shapes containing osmiophilic granules and remnants of contractile material were present (Figure 5D).

**Figure 7:**
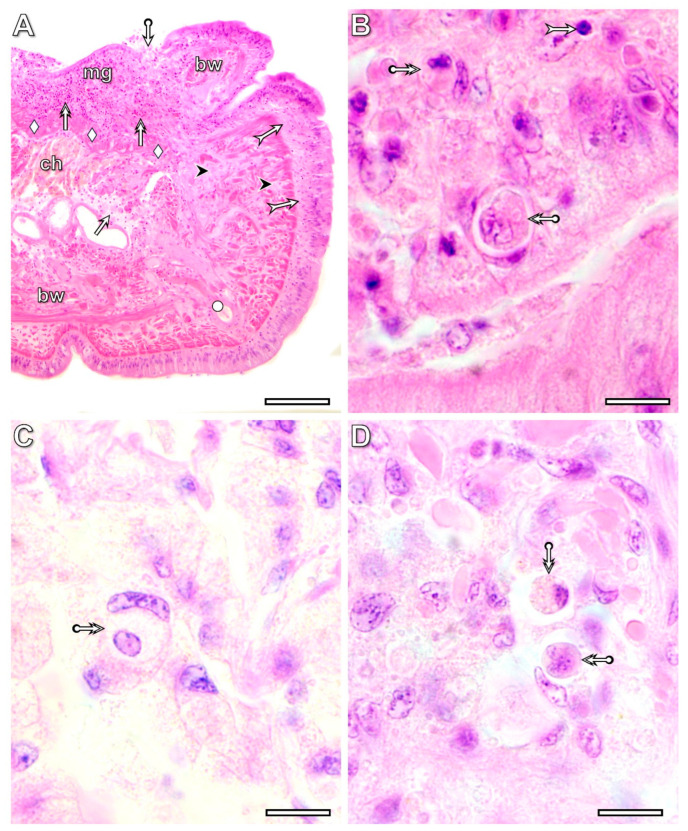
Low magnification (**A**) shows the fusion of the midgut and body wall tissues, as well as the accumulation of cells between the midgut muscle and epithelial layers by the 24th postoperative hour. High magnification (**B**–**D**) reveals the heterogeneous morphology of the accumulated cells. Note the occurrence of distinct types of the cell-in-cell phenomenon within this cell mass. Labeling: bw: body wall, ch: chloragogenous tissue, mg: midgut, deltoid: muscle layers of the midgut, arrow: coelomocytes, double arrows: accumulated cells in the midgut wall, forked arrow: dedifferentiated epithelial cells, arrowhead: dedifferentiated muscle cells, bullet double arrow: cell-in-cell phenomenon, circle: chaete. Scale bars: (**A**): 100 μm, (**B**–**D**): 10 μm.

**Figure 8:**
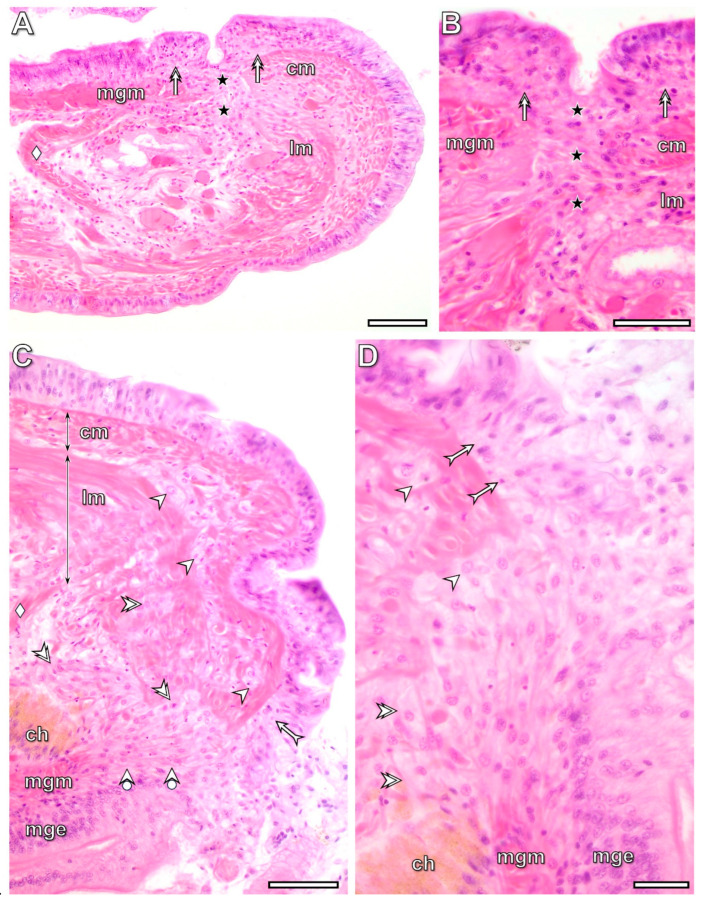
Histological characteristics of the lateral (**A**,**B**) and dorsal (**C**,**D**) body wall at the 24th postoperative hour. Note that the fusion of both epithelial and muscle tissues of the body wall and midgut is complete in the lateral part of the segment. In contrast, in the dorsal part, the epithelial organization is not yet fully developed. Labeling: cm: circular muscles, lm: longitudinal muscles, ch: chloragogenous tissue, mge: midgut epithelium, mgm: midgut muscles, asterisk: fused tissues, arrowhead: dedifferentiated cells in the circular muscles, double arrowhead: dedifferentiated cells in the longitudinal muscles, forked arrow: dedifferentiated epithelial cells. Labeling: ch: chloragogenous tissue, cm: circular muscles, lm: longitudinal muscles, mgm: midgut muscles, mge: midut epithelium, deltoid: dissepiment, double arrow: dedifferentiated epithelial cells, arrowhead: dedifferentiated circular muscle cells, double arrowhead: dedifferentiated longitudinal muscle cells, asterisk: fusion of the midgut and body wall tissues, bullet arrow: dedifferentiated epithelial cell, forked arrow: fused muscle fibers of the midgut and body wall. Scale bars: (**A**–**C**): 100 μm, (**D**): 25 μm.

By the 24th postoperative hour, the body wall and midgut tissues—including both epithelium and muscle layers—had fused with each other, and the fusion zones were clearly identifiable in various regions of the regenerating segment (Figure 7 and Figure 8). Distinct differences were observed between the organization of the fused tissues in the ventral part of the segment versus the lateral and dorsal parts. In the ventral region, a dense mass of cells accumulated beneath the midgut epithelium (Figure 7A,B). These cells had small, compact, basophilic nuclei surrounded by a thin rim of cytoplasm containing various granular structures. Among these cells, a previously undescribed cytological phenomenon occurred frequently: one cell engulfed another living cell. The engulfed cells exhibited markedly different morphologies; some showed clear signs of programmed cell death, whereas others appeared viable (Figure 7B–D). Another histological difference between the fusion zones was the more advanced reorganization of the ventral part—which contains the ventral nerve cord—compared with the lateral and dorsal parts. While the muscle layers of the midgut and lateral (Figure 8A,B) and dorsal (Figure 8C,D) body wall had completely fused, epithelial differentiation was not yet complete.

**Figure 9:**
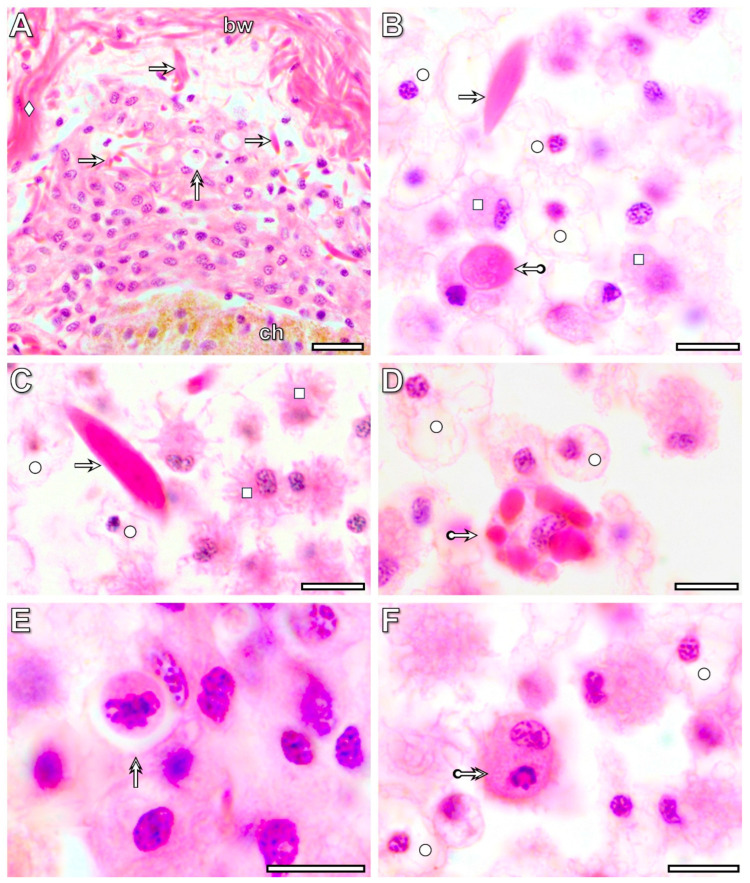
Light micrograph of coelomocytes accumulated in the intact segments located anterior to the transected segment of the regenerating *E. andrei* at the 3rd postoperative day (**A**). Note that the light microscopic characteristics of the coelomocytes differ from those of the dedifferentiated tissue cells that close the wound (see Figure 2, Figure 7 and Figure 8). Some amoebocytes engulf degenerating muscle fibers (**B**–**D**) and, unexpectedly, some other coelomocytes as well (**E**,**F**). The fate of the engulfed cells can vary, as some appear normal (**E**), while others seem to be undergoing apoptosis (**F**). Labeling: bw: body wall, ch: chloragogenous tissue, arrow: muscle fibers, double arrow: cell-in-cell phenomenon, circle: eleocytes, square: amoebocyte, bullet arrow: engulfed muscle fibers in amoebocyte, deltoid: dissepiment. Scale bars: (**A**): 25 μm, (**B**–**F**): 10 μm.

By the 3rd postoperative day, a significant accumulation of coelomocytes (amoebocytes and eleocytes) was detected in intact segments located anterior to the transected one. They formed cell masses of various sizes together with detached muscle fibers in the coelom (Figure 9A). Eleocytes were easily distinguished from other cells by their vacuole-rich, pale-staining cytoplasm, whereas identifying amoebocytes required more detailed observations. The form and position of their nucleus, cytoplasmic staining properties, and presence of pseudopods served as cytological markers (Figure 9B,C). Amoebocytes frequently engulfed muscle fibers (Figure 9D) and occasionally other coelomocytes as well. The fate of the engulfed cells varied: some appeared normal (Figure 9E), whereas others showed apoptotic features (Figure 9F).

### 3.4. Ultrastructural Alterations of the Wound Closing Tissue

**Figure 10:**
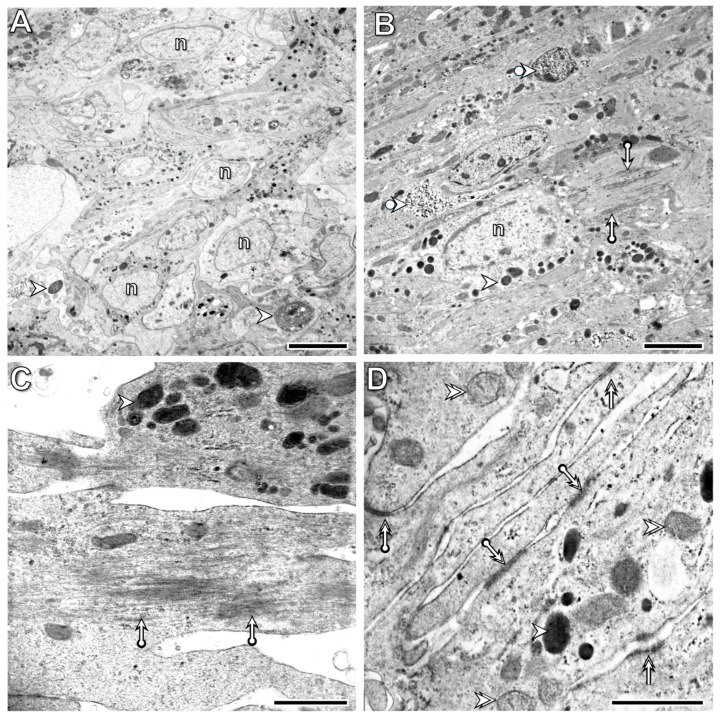
Ultrastructural characteristics of the white tissue formed at the wound boundary at the 3rd (**A**,**B**) and 24th postoperative hours (**C**,**D**). The lower magnification (**A**) shows tightly attached blast-like cells with an euchromatic nucleus and several osmiophilic (lysosome-like) structures in the cytoplasm. Note that no coelomocytes are present in this cell mass. Higher magnification reveals remnants of contractile proteins in the cytoplasm of some dedifferentiated cells (**B**), which are also observed in dedifferentiated cells at the 24th postoperative hour (**C**). Dedifferentiated cells display several anchoring (desmosome-like) and communicating (gap junction-like) cell junctions, characteristic of differentiated muscle fibers. Labeling: n: nucleus, arrowhead: lysosome-like structure, double arrowhead: swollen mitochondrion, bullet arrowhead: intracellular collagen fibers, bullet arrow: contractile proteins, double arrow: desmosome-like cell junction, double bullet arrow: gap junction-like cell junction. Scale bars: (**A**): 5 μm, (**B**): 2 μm, (**C**,**D**): 1 μm.

The cell mass that appeared at the wound edge by the 3rd postoperative hour (Figure 2C) consisted of tightly attached blast-like cells with a large, euchromatic nucleus and a thin cytoplasmic rim containing several osmiophilic (lysosome-like) structures (Figure 10A). No coelomocytes were present among these cells. Higher magnification revealed remnants of contractile substances and collagen fibrils in the cytoplasm of some cells (Figure 10B). By the 24th postoperative hour (Figure 10C,D), the shape of the cells had characteristically changed, but they still contained some contractile substances, and desmosome- and gap junction-like structures were frequently observed between their cellular processes.

### 3.5. Distribution Pattern of Collagen Fibers in the Original and Regenerating Parts of the Body Wall

**Figure 11:**
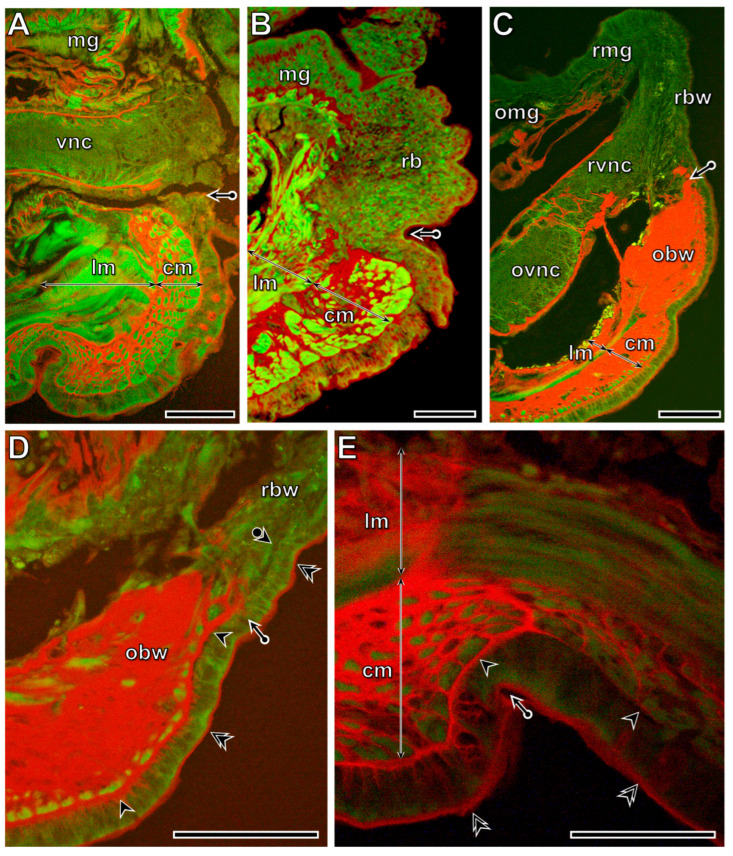
Distribution pattern of collagen deposition (red staining) in tissues of freshly operated (**A**) and regenerating specimens of *E. andrei* at the 1st postoperative day (**B**), 1st postoperative week (**C**,**D**), and 5th postoperative week (**E**). In freshly operated specimens, as in the intact segment, some collagen bundles are located around the muscle fibers of the circular muscle layer. Reorganization of collagen fibers in the transected segment and the formation of collagen-free new tissue are characteristic features of regeneration. By the 5th postoperative week (**E**), the circular and longitudinal muscle layers can already be distinguished in the transected segment; however, significantly thicker collagen bundles surround the circular muscle fibers compared to the control. Moderate collagen deposition is also observed in the muscle layers and the basement membrane. Labeling: cm: circular muscles, lm: longitudinal muscles, mg: midgut, vnc: ventral nerve cord, obw: original body wall, rbw: regenerating body wall, omg: original midgut, rmg: regenerating midgut, ovnc: original ventral nerve cord, rvnc: regenerating ventral nerve cord, rb: regeneration blastema, bullet arrow: site of the transection, arrowhead: original basement membrane, double arrowhead: cuticle, bullet arrowhead: developing basement membrane. Scale bars: (**A**–**D**): 100 μm, (**E**): 50 μm.

Transection induced a rapid realignment of the collagen fiber distribution pattern in the body wall tissues, which was recognizable by the 1st postoperative day, when the regeneration blastema had formed. At the boundary of the circular muscle layer, collagen fibers were markedly deposited, whereas no such deposition occurred in the longitudinal muscles (Figure 11B). By the 1st postoperative week, a prominent difference in collagen deposition was observed between the original and regenerating tissues. In the regenerating tissues, except for the cuticle, no collagen fibers were detected. The most conspicuous collagen deposition occurred in the body wall of the transected segment, where the circular and longitudinal muscle layers could not be distinguished (Figure 11C). However, the developing epithelial and muscle layers were already separated from each other, and no collagen was deposited into the developing basement membrane (Figure 11D). By the 5th postoperative week (Figure 11E), the circular and longitudinal muscle layers were distinguishable again in the transected segment; however, significantly thicker collagen bundles surrounded the circular muscle fibers compared with the original muscle layer. Moderate collagen deposition was also present in the muscle layers and in the basement membrane.

### 3.6. Ultrastructural Characteristics of the Regenerating and Original Body Wall Tissues

**Figure 12:**
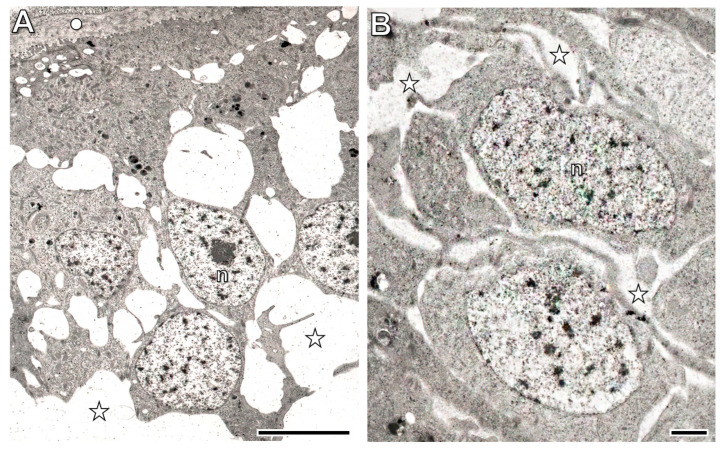
Ultrastructure of the prospective body wall in the regeneration blastema on the 1st postoperative week. Note that blast cells are present in both the prospective body wall epithelium (**A**) and muscle layers (**B**), and no collagen is observed in the extracellular spaces. Labeling: circle: cuticle, n: nucleus, asterisk: extracellular space. Scale bars: (**A**): 2 μm, (**B**): 1 μm.

In the prospective body wall tissues identified at the 1st postoperative week (Figure 11C), a thin cuticle containing some collagen fibers appeared on the surface of the differentiating epithelial cells, which still exhibited several blast-like characteristics. These small cells had a large nucleus and were connected to each other by cellular processes, with extended extracellular spaces located between them (Figure 12A). The prospective muscle layers consisted of blast-like cells, and no collagen fibers were observed in the extracellular spaces (Figure 12B).

**Figure 13:**
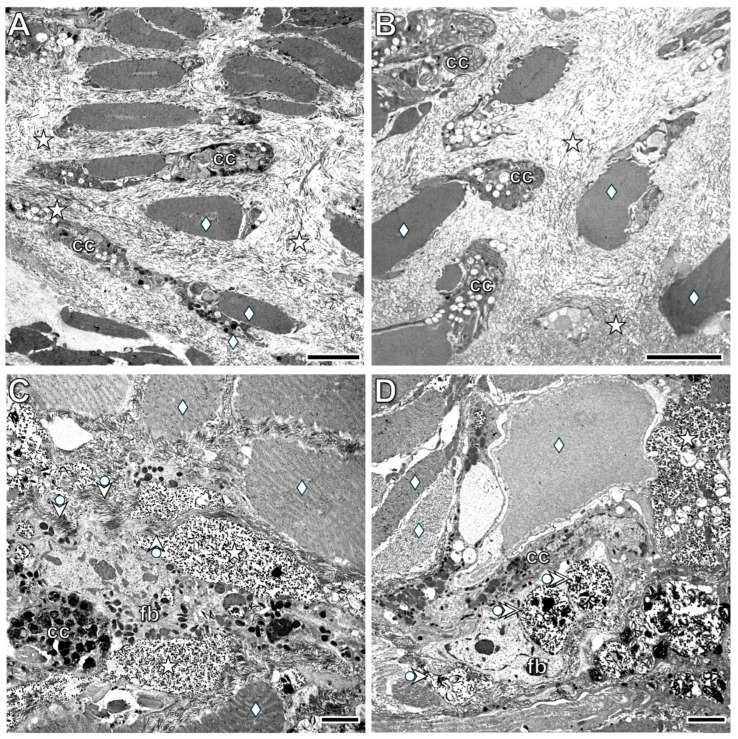
Ultrastructural characteristics of the muscle layer in the original parts of the transected body wall located in the farther (**A**) and in the nearer part (**B**) of the segment to the transection at the 1st postoperative week. Note that among the dark muscle fibers, extended extracellular spaces densely packed with collagen are observed. In the nearer part of the segment, even more extensive extracellular spaces are present between the muscle fibers. The intimate connection between coelomocytes and muscle fibers indicates communication between them. In distinct regions of the muscle layers, a high number of fibroblasts, characterized by extremely intense collagen production, are found (**C**,**D**). Their morphological characteristics are similar to those of vertebrate fibroblasts. Labeling: cc: coelomocyte, fb: fibroblast, deltoid: muscle fiber, asterisk: collagen deposition in the extracellular spaces, bullet arrowhead: exocytosed collagen fibrils, double bullet arrowhead: intracellular accumulation of collagen fibrils. Scale bars: (**A**,**B**): 5 μm, (**C**,**D**): 2 μm.

Electron microscopic examinations of the original parts of the transected segment supported the light microscopic results. Dark muscle fibers were present in both the circular and longitudinal muscle layers; they were densely packed in the farther part of the segment and sparsely packed in the region closer to the transection. Extended extracellular spaces filled with collagen fibers were located between the muscle fibers, showing close association with immigrating coelomocytes (Figure 13A,B). In distinct regions of the muscle layers, a high number of fibroblasts—characterized by extremely intense collagen production—were found (Figure 13C,D). Their morphological characteristics resembled those of vertebrate fibroblasts.

**Figure 14:**
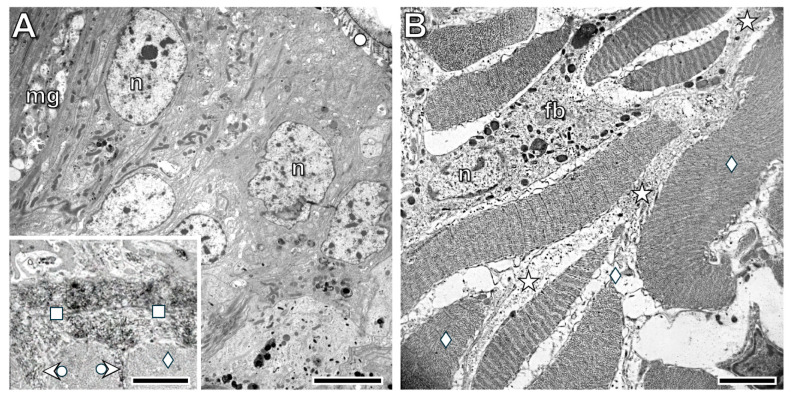
Ultrastructure of the regenerated body wall epithelium (**A**), circular muscles (insert (**A**)), and longitudinal muscle layer (**B**) at the 5th postoperative week. Note that the structure of the epithelium is similar to that of the control, with a collagen-rich cuticle and basement membrane being characteristic features. Some collagen deposition is also observed between the circular muscle fibers (insert (**A**)). In the longitudinal muscles, only weak collagen deposition is present; however, fibroblasts are already present between the muscle fibers. Labeling: fb: fibroblast, mg: mucous gland, n: nucleus, deltoid: muscle fiber, circle: cuticle, square: basement membrane, bullet arrowhead: collagen deposition between circular muscle fibers, asterisk: collagen deposition between longitudinal muscle fibers. Scale bars: (**A**): 5 μm, insert (**A**): 2 μm, (**B**): 2 μm.

No significant changes in the organization of the original and regenerating body wall tissues were observed by the 3rd postoperative week. By the 5th postoperative week, the structure of the epithelium in the regenerating body wall resembled the original organization; a collagen-rich cuticle and basement membrane were characteristic features (Figure 14A). Some collagen deposition was also present between the circular muscle fibers (insert of Figure 14A). In the longitudinal muscle layer, only weak collagen deposition was detected; however, some fibroblasts were already present between the newly formed muscle fibers (Figure 14B).

## 4. Discussion

There is a long-standing debate about various aspects of segment regeneration in earthworms, including the early stages of the process (e.g., wound closure and tissue dedifferentiation), the basis of its scar-free nature, and the fidelity of regeneration. Our results show that segment regeneration starts with extremely rapid and precise wound healing, which does not leave scar tissue between the original and renewed tissues. Fast wound closure is a critical step in the regeneration process, protecting the injured tissues against microbial invasion and preventing loss of body fluids.

Previously, the coelom-closing cell plug has been identified as a grouping of coelomocytes [43]. Moreover, it has been claimed that epidermal basal cells do not directly contribute to wound epithelialization and that there is little evidence for body wall muscle dedifferentiation [44,45]. In contrast, a later study suggested that the longitudinal muscle layer of the body wall is the primary source of blastemal cells [46]. In the present study, detailed histological and cytological investigations revealed a rapid dedifferentiation of the epithelium and obliquely striated muscle fibers of the body wall, resulting in the formation of a high number of epithelioblast-like and myoblast-like cells that contribute to wound closure and blastema formation. Based on our findings and a careful review of previous studies, we suggest the following: (1) the grouping coelomocytes as coelom-closing cells may have been misinterpreted [43]; (2) epithelium and muscle dedifferentiation may have been overlooked due to sampling timing [44,45]; and (3) the role of circular muscles and the epithelium may not have been recognized [46]. Taken together, we propose a new, comprehensive model for the early stage of segment regeneration. First, the body wall and midgut primarily contribute to the wound closure. Second, the wound closing plug consists of epithelioblast-like and myoblast-like cells derived from the dedifferentiation of the epithelium and both muscle layers of the body wall. Third, coelomocytes phagocytose damaged and detached muscle fibers but do not play a direct role in wound closure or blastema formation; however, their bioactive compounds may influence dedifferentiation, proliferation, and redifferentiation of various tissue cells. Fourth, the VNC likely has only a modulatory role, which requires further investigation.

It is noteworthy that some open questions regarding the early stages of segment regeneration remain unresolved. First, we observed a remarkable heterotypic cell-in-cell phenomenon in the granular tissue and in some cases in coelomocytes. The cell-in-cell phenomenon is a well-recognized feature in pathology (e.g., Rosai–Dorfmann disease) and cancer research (reviewed by refs. [47,48,49]). Its exact physiological role is not fully understood, but it may play key roles in inflammation and tissue homeostasis by eliminating damaged cells, regulating tissue turnover, and contributing to immune modulation. Speculatively, we propose that the observed phenomenon is phagoptosis in the granular layer and emperipolesis in the case of coelomocytes, suggesting that the cell-in-cell phenomenon likely plays an important role in immune surveillance and tissue remodeling during early segment regeneration. Future studies should aim to investigate the cell-in-cell phenomenon in both invertebrate and vertebrate regeneration models in detail. Second, pattern recognition receptors are expected to play key roles in tissue recognition between the body wall and midgut during wound closure. A previous study examined their expression during both anterior and posterior segment regeneration at 1, 2, 3, and 4 weeks post-amputation and found significant increases, decreases, or no changes after the first week, depending on the receptor studied [50]. Given that the early stage of segment regeneration involves tissue recognition and wound closure, and that pattern recognition receptors can respond rapidly to tissue damage or damage-associated molecular patterns, measuring their expression at one week (or later) post-amputation likely captures later immune-modulatory or tissue-remodeling effects rather than the immediate recognition events. Future studies should also include early time-point analyses to distinguish immediate wound-sensing roles from secondary immune responses (i.e., to differentiate immune vs. regenerative roles).

An unexpected result of our experiments was the absence of collagen fibers in the regeneration blastema and differentiating tissues (cuticle, mussel layers) at early phases of the regeneration. Collagens, a family of trimeric extracellular matrix molecules, are synthesized from early embryonic to senescent stages with various functions, including morphogenesis, strengthening, tissue maturation, maintenance, adaptation, and repair [51]. During both embryogenesis and physiological tissue regeneration, collagens serve as a substrate for cellular attachment and promote cellular proliferation, growth, and differentiation [52]. Their presence in the extracellular matrix is necessary for the wound healing processes of animals from sponges to mammals [24]. Even on the fifth postoperative week, collagen was deposited only in the cuticle, whereas the basal membrane and the circular muscle layer contained only minimal collagen. In mammalian fetal wound healing, type III collagen is rapidly deposited, forming a fine, flexible network, while type I collagen appears later, and the I:III ratio remains low, supporting regenerative, scarless repair. In adult wounds, type III collagen is initially deposited during the granulation phase but is rapidly replaced by type I collagen during remodeling. The collagen fibers in adults become thick, aligned, and densely packed in parallel bundles, and type III collagen declines as remodeling proceeds, resulting in a high collagen I:III ratio (reviewed by refs. [53,54]). With other mechanisms (e.g., inflammation), this shift from type III to type I underlies the transition from regenerative fetal healing to fibrotic adult repair. Interestingly, both the circular and longitudinal muscle layers of the original segment adjacent to the regenerating one contained thick, aligned collagen bundles during the third week. However, by the fifth week, collagen was absent in the longitudinal layer and formed a fine, random network in the circular layer, indicating a dynamic change in collagen deposition within the neighboring original segment. We propose that these dynamic changes in collagen serve a scaffold function during regeneration, providing a framework for migrating dedifferentiated cells and maintaining tissue integrity.

The absence of collagen fibers in the early blastema and differentiating tissues suggests that regeneration proceeds in a soft, permissive extracellular matrix, allowing dedifferentiated cells to migrate and proliferate freely. Hence, collagen appears to function primarily as a secondary structural scaffold rather than as an initial framework, consistent with regenerative, scarless-like healing. The delayed, selective deposition of collagen in the cuticle by the fifth postoperative week likely reflects the onset of mechanical stabilization rather than a fibrotic response (i.e., collagen is incorporated later for structural reinforcement). Dynamic remodeling in the neighboring segment further supports this interpretation. The presence of thick, aligned collagen in the adjacent segment by the third week indicates that mature tissues maintain a stable extracellular matrix, while the subsequent shift in fiber organization between circular and longitudinal muscle layers by the fifth week highlights region-specific extracellular matrix remodeling that may guide regenerating cells.

The delayed collagen deposition in the regenerating body wall tissues of earthworm shares some properties with scar-free, regenerative wound healing observed in certain mammals, such as spiny mice (*Acomys* spp.) and naked mole rats (NMRs). The extracellular matrix of the newly formed tissues in spiny mice contains only small amounts of collagen, especially type I collagen; instead, tenascin, fibronectin, and ostepontin predominate, acting as pro-proliferative and pro-regenerative provisional matrix components [55]. In NMRs, low-scar wound healing has been attributed to a low type I to type III collagen ratio, elevated fibronectin expression, and a high hyaluronan content in the extracellular matrix [56]. Scar-free (regenerative) wound healing in both models has been suggested to share morphological and biochemical similarities with scarless fetal wound healing in mammals [55,56]. Overall, the pattern of delayed collagen deposition observed during earthworm segment regeneration suggests that certain steps of this process resemble the regenerative wound healing mechanisms of spiny mice, NMRs, and scarless fetal wound healing in vertebrates. Because experiments involving mammals are considerably more costly than those using invertebrates, and because both wound healing and regeneration proceed more rapidly in earthworms, earthworm tail regeneration represents a suitable model for investigating the morphological and biochemical basis of tissue differentiation during regeneration. The prospective results may contribute to a better understanding of tissue and organ regeneration and help elucidate its ontogenetic and phylogenetic significance.

Earthworms have at least two distinct types of collagen: a cuticular-type synthesized by the body wall epithelium and a muscular-type located in the muscle layers of the body wall, perineural sheath, and alimentary canal [57,58,59,60,61]. A recent study also identified a novel collagen-like peptide, col4a1, in *E. andrei* and demonstrated its significant effects on wound healing both in vitro *and* in vivo, including enhanced viability, proliferation, and collagen deposition [62]. The identification of a novel fibroblast-type collagen suggests that, like vertebrates, earthworms have distinct cellular sources of collagen with potentially divergent roles in tissue organization and repair, revealing greater structural and functional complexity in annelid extracellular matrices than previously recognized.

## 5. Conclusions

In summary, our study reveals that earthworm segment regeneration is a highly coordinated, scarless process characterized by rapid wound closure, extensive dedifferentiation of epithelial and muscle cells, and the formation of a blastema in a permissive extracellular matrix. Coelomocytes appear to function primarily in debris clearance and immune modulation rather than directly contributing to blastema formation. The delayed and region-specific deposition of collagen underscores its role as a secondary structural scaffold, supporting tissue integrity and guiding regenerating cells without impeding cellular migration and proliferation. These findings suggest that earthworm regeneration shares key mechanistic features with scarless fetal wound healing in vertebrates, including a soft early extracellular matrix and temporally controlled collagen incorporation.

From an evolutionary aspect, our findings highlight the conserved role of extracellular matrix dynamics and dedifferentiation in tissue regeneration across metazoans. The identification of distinct collagen types and dynamic extracellular matrix remodeling in earthworms provides new insights into how structural and biochemical cues coordinate regeneration. Understanding these mechanisms might also inspire novel strategies for promoting scarless repair and enhancing tissue regeneration in mammals. For instance, mimicking the early soft extracellular matrix environment observed in earthworms, combined with controlled temporal deposition of collagen, could reduce fibrosis and promote scarless healing. Similarly, stimulating dedifferentiation or transient plasticity in mammalian cells might improve regenerative outcomes in tissues that normally have limited repair capacity. Finally, understanding the immunomodulatory role of coelomocytes may guide approaches to modulate inflammation during wound healing, creating a more permissive environment for regeneration.

## Data Availability

All micrographs and figures generated during the histochemical and ultrastructural investigations, which are necessary for interpreting the results and supporting the claims, are fully included in the manuscript.

## Abbreviations

The following abbreviations are used in this manuscript:

H&E	hematoxylin and eosin
RB	regeneration blastema
